# Three-Dimensional Scanning Accuracy of Intraoral Scanners for Dental Implant Scan Bodies—An Original Study

**DOI:** 10.3390/medicina59112037

**Published:** 2023-11-19

**Authors:** Cristian Rotaru, Elena Adina Bica, Cristian Butnărașu, Mihai Săndulescu

**Affiliations:** 1 Doctoral School, Carol Davila University of Medicine and Pharmacy, 020021 Bucharest, Romania; cristian.rotaru@drd.umfcd.ro; 2 Faculty of Dental Medicine, Titu Maiorescu University of Medicine, 040441 Bucharest, Romania; 3 MINEC—MegaGen International Network of Education & Clinical Research, 030925 Bucharest, Romania; 4 Department of Implant-Prosthetic Therapy, Faculty of Dentistry, Carol Davila University of Medicine and Pharmacy, 020021 Bucharest, Romania

**Keywords:** intraoral scan, digital dentistry, scanning accuracy

## Abstract

*Background and Objectives*: With the increased trend towards digitalization in dentistry, intraoral scanning has, to a certain extent, replaced conventional impressions in particular clinical settings. Trueness and precision are essential traits for optical impressions but have so far been incompletely explored. *Materials and Methods*: We performed a study to evaluate the differences in the three-dimensional spatial orientations of implant analogs on a stone cast when using an intraoral scanner compared to a dental laboratory scanner. We assessed the deviation of the intraoral scans compared to the laboratory scan for three standardized implant measurement plans and compared these results with control scans of the neighboring natural teeth. *Results*: We found no statistically significant correlation between the measurements at the scan body level and the landmarks chosen as controls on the neighboring natural teeth (*p* = 0.198). The values for the implant scans presented wider variation compared to the control scans. The difference between the implant and the control planes ranged from −0.018 mm to +0.267 mm, with a median of −0.011 mm (IQR: −0.001–0.031 mm). While most values fell within a clinically acceptable margin of error of 0.05 mm, 12.5% of the measurements fell outside of this acceptable range and could potentially affect the quality of the resulting prosthetic work. *Conclusions*: For single-unit implant-supported restorations, intraoral scanning might have enough accuracy. However, the differences that result when scanning with an intraoral scanner may affect the quality of prosthetic work on multiple implants, especially if they are screw-retained. Based on our results, we propose different adaptations of the prosthetic protocol to minimize the potential effect of errors that may occur during the digital workflow.

## 1. Introduction

“Digital dentistry” was mentioned for the first time in PubMed in 1999 and has become increasingly integrated in the field of fixed prosthodontics [1]. The digital revolution is changing the world, and new digital tools in dentistry have become almost indispensable in our daily work. Nowadays, we can benefit from the most advanced technologies, such as intraoral and facial scanners, cone-beam computed tomography systems (CBCTs), technologies that analyze jaw movements, CAD/CAM technology, 3D printing and digital planning [2].

Few innovations over the past 20 years have changed dentistry as much as the introduction of dental scanners. This invention represents the first step towards digitalization, being first used in the dental laboratory to scan a stone cast, and later on the introduction of intraoral scanners allowed clinicians to skip an otherwise unavoidable step (the conventional impression) by replacing it with a direct intraoral scanning procedure [2]. 

Even though the analogue impression techniques have proven their efficiency over the last decades—both for teeth and dental implants—the optical impression technique brought many advantages, such as less patient discomfort, better communication with the dental technician, and reduction in working time and therefore overall treatment costs [3]. 

Implant dentistry as a treatment modality is nowadays a routine therapeutic option and has become crucial for restoring aesthetics and masticatory function in oral rehabilitation. Dental implants are functionally ankylosed to the bone without periodontal ligament support over the direct contact between the living bone and the implant, as Albrektsson et al. [4] defined the osseointegration process. However, the long-term success of dental implants relies on the way the passive framework fits, which is a core principle with respect to the outcome [4,5]. 

A thorough understanding of the role that mechanical stress plays is crucial, as it may lead to bone loss if there is a lack of a passive fit between dental implants and fixed dental restoration; therefore, we emphasize the importance of an adequate and uniform distribution of the occlusal forces to the bone–implant interface [5].

The first prosthetic step in achieving the passive fit is through the impression, in which the three-dimensional positions of dental implants are recorded and transferred. Any inaccuracy in this process will increase the stress to the implants and prosthetic restoration and may result in mechanical and biological complications [6,7]. 

In the conventional workflow, the main goal is to obtain a precise implant master cast by using precise materials and techniques for the impression taking and stone cast pouring. There are some listed factors that could affect the accuracy of implant impressions, such as the impression technique used (open-/closed-tray technique and splinting impression copings), surface treatment of impression copings, the angulation and the depth of implants, and the connection type [8].

On the other side, intraoral scanners are certainly user-friendly tools that subserve daily clinical practice. One of the advantages of optical impressions is the ability to directly capture all the dental arch information of the patient, transfer it into a 3D virtual model and e-mail the STL file directly to the dental laboratory. The success of treatment relies on the accuracy of this procedure; therefore, trueness and precision are highly required [9,10]. 

Trueness is defined as the “closeness of agreement between the expectation of a test result or a measurement result and a true value” [9,10]. Ideally, the digital impression (the measurement) should be able to match the intraoral situation (the real value) as closely as possible. The only means of calculating the trueness of an intraoral scanner is by superimposing a digital impression of a scanned object on a reference scan of the same object obtained with a powerful industrial machine (an industrial optical scanner). After the overlapping of these scans, powerful reverse-engineering software can be used in order to determine deviations mathematically [11].

Precision is the “closeness of agreement between indications or measured quantity values obtained by replicated measurements on the same objects under specified conditions” [9,10]. Precision can be calculated more easily by overlapping different scans of the same object obtained with the same IOS device at different times and then evaluating the deviations at the micrometric level once more [9,10].

There are many factors that might compromise the accuracy of this procedure, including scanning technology, the experience of the clinician, scanning technique, the design and material of the scan bodies, etc. In vivo, limited mouth opening, an oversized tongue and hypersalivation should also be considered as accuracy-influencing factors [12]. 

Therefore, the aim of our study was to evaluate the differences in the three-dimensional spatial orientations of implant analogs on a stone cast (therefore not in vivo) when using an intraoral scanner compared to a dental laboratory scanner. 

The null hypothesis was that no differences would be found between digital impressions and conventional open-tray impressions using vinyl polysiloxane (VPS) impression materials. 

## 2. Materials and Methods

The study is based on a comparison of the accuracy of the intraoral scanner and the laboratory scanner, the latter being considered the gold standard at the moment. For this pilot study, we randomly chose 3 cases provided by a dental clinic in Bucharest, Romania, in which we used stone casts to make a scan with a laboratory scanner as a reference and other scans with an intraoral scanner in 3 different planes at the level of implant scan bodies.

All 3 master casts used in this study were obtained through classic impressions—open-tray impressions which had been previously sent to a dental laboratory to obtain the stone cast.

**Figure 1:**
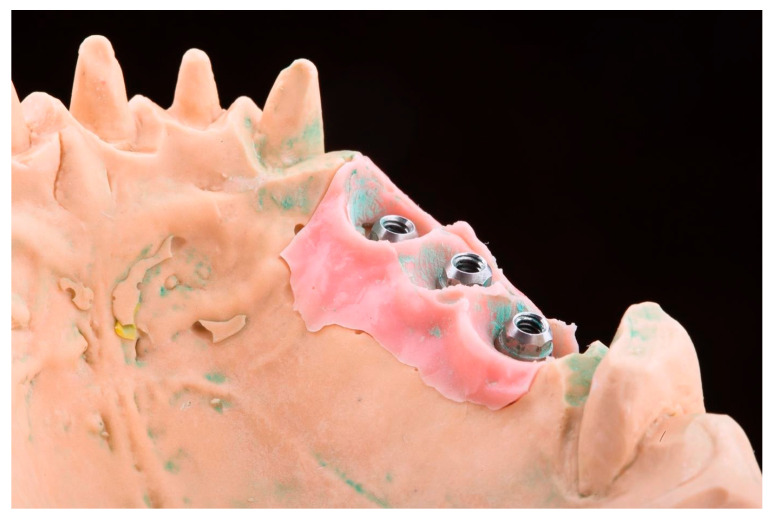
The master cast with a gingival mask overlaying the implant analogs. First case—partial edentulous space in the 2nd quadrant, with 3 dental implants on positions 2.4, 2.5 and 2.6.

The first case was represented by a partial edentulous ridge in the 2nd quadrant, with 3 dental implants on positions 2.4, 2.5 and 2.6, with Octa abutments placed on the implants. A stone cast with a silicone gingival mask overlaying the implant analogs was used for the study measurements (Figure 1).

**Figure 2:**
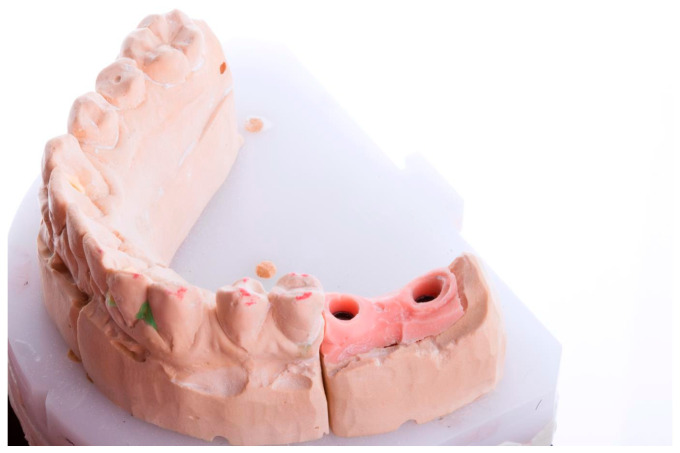
Case 3. The master cast obtained after an implant-level impression technique.

The 2nd case had a free-end edentulous space in the 1st quadrant, with 2 implants on positions 1.6 and 1.7, and the last case included in our study had a free-end edentulous space in the 3rd quadrant with 2 implants on positions 3.6 and 3.7 (Figure 2). For all three cases, Megagen Anyridge implants had been used (MegaGen, Daegu, Republic of Korea). 

All stone casts were scanned using an intraoral scanner (Trios 3, 3Shape, Copenhagen, Denmark). The scanning protocol started on the Patients page of 3Shape Software, after which we created the patient file, then selected the implant position, the type of implant, the connection and the restoration type, namely, screw-retained splinted crowns. We moved on to scanning the soft tissues around the implants and the neighboring teeth. The digital impression was obtained using an “S” scan path; the scanner tip followed the entire arch, starting from the right side on the occlusal surfaces, continuing with the vestibular surfaces of the teeth and finishing with the oral surfaces to ensure good data coverage of all surfaces and to follow the recommended scan strategy [13]. The 2nd stage consisted of scanning the scan bodies screwed on the implants with the hexagonal half placed towards the buccal part of the model. The data were stored as an STL file. In order to establish the accuracy of the 3D spatial orientations of the scan bodies on our master casts, we performed 5 scans for each case. 

**Figure 3:**
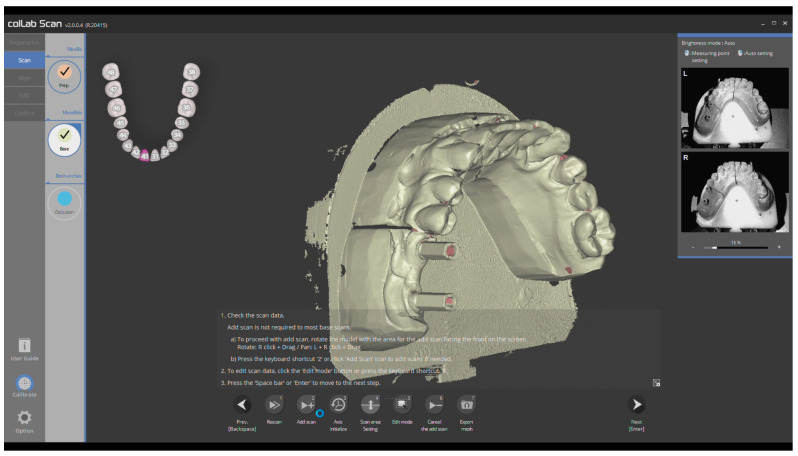
Digital impression of the 3rd master cast using Medit T300.

The master casts were then sent to the dental laboratory and digitized using a high-resolution lab scanner (Medit T300, Medit Corp, Seoul, Republic of Korea) and COLLAB SCAN software (Medit Corp, Republic of Korea) (Figure 3). After the acquisition of digital impressions, the digital volumes were exported as STL files and superimposed on the STL files acquired by the intraoral scanner. A total of 5 superimposed files for each model were obtained in Exocad Plovdiv software (Exocad GmbH, Darmstadt, Germany) by using the “best fit matching” function at 3 reference points on the digital casts.

**Figure 4:**
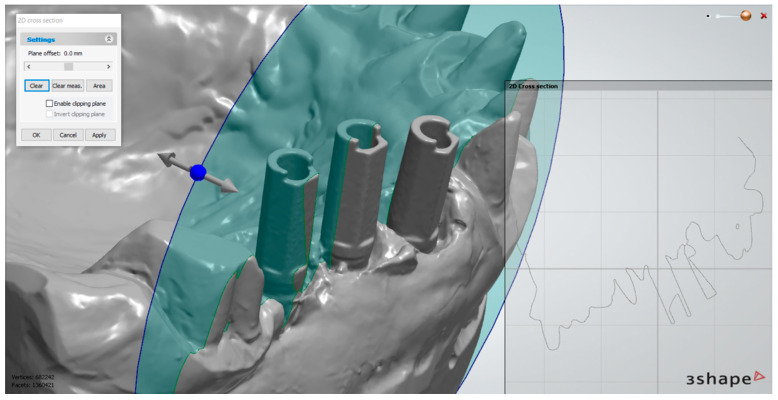
First plane of reference—shown at the level of the middle implant. After establishing the plane, the measurements were performed in “2D Cross section”.

**Figure 5:**
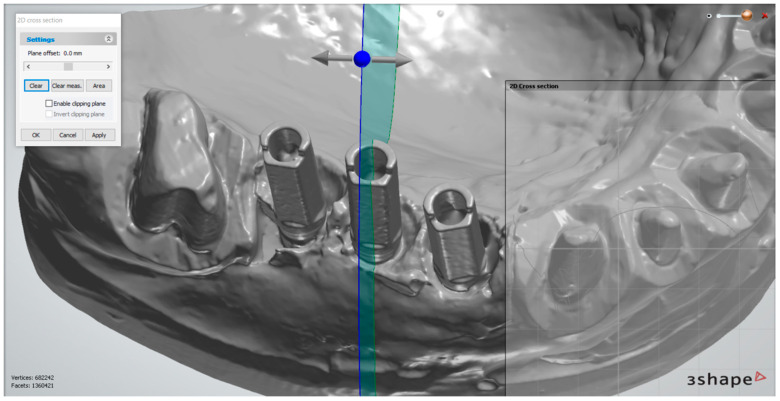
The second plane of reference—passing through the central axis of the abutment, perpendicular to its central side.

**Figure 6:**
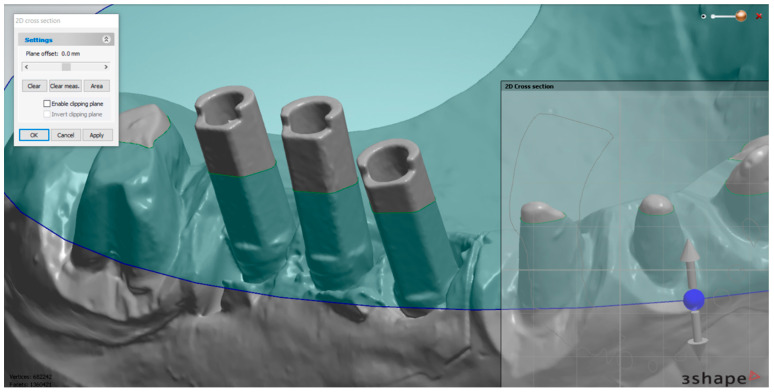
The third plane of reference—perpendicular to the axis of the abutment.

The measurements were performed in the superimposed scans at the level of the scan abutments using the software 3Shape 3D Viewer (3Shape, Copenhagen, Denmark). To assess the reproducibility of the measurements made at each implant, we defined 3 standardized sections. The first plane of reference was parallel to the central side of the scanning abutment, which passed through its central axis (Figure 4). The second plane was perpendicular to its central side, intersecting the central axis (Figure 5). The third plane was perpendicular to the vertical axis of the scanning abutment, in the upper part of the abutment, in the proximity of its index (Figure 6). 

**Figure 7:**
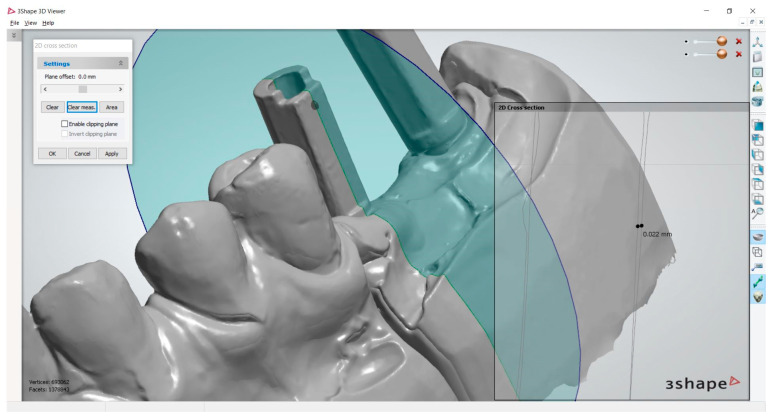
Example of a measurement made in the 3Shape 3D Viewer software. In this case, the difference noted was 0.022 mm.

Two control measurements were also made at the level of the adjacent natural teeth in order to eliminate the errors that may occur after an inaccurate superimposition of intraoral scans (the in-office scan and the laboratory scan) (Figure 7). For the purpose of the current analysis, we report the average values of the two control measurements.

The results were analyzed statistically using the IBM SPSS Statistics v25 software (IBM Corp, Armonk, NY, USA). Variables are reported in mm and represent the measured deviation of the intraoral scan compared to the reference laboratory scan for each implant plane and for the average values for all three implant planes. To ascertain whether the continuous variables had a parametric or a non-parametric distribution, we applied the Shapiro–Wilk test. For the variables with a non-parametric distribution, the median and the interquartile range (IQR) are presented, and we used raincloud plots to depict the raw data and the data distribution, along with the IQR and the median as a boxplot. To identify if there were significant statistical correlations between the non-parametric continuous variables, Spearman correlation analysis was used. All analyses were two-tailed. Values below *p* < 0.05 were considered statistically significant. 

## 3. Results

We first assessed the deviation (in mm) of the intraoral scans compared to the laboratory scan for each of the three standardized implant measurement plans. The medians were 0.031 mm, 0.025 mm and 0.037 mm for the first, second and third implant planes, respectively (Table 1). We analyzed if there was a statistically significant correlation between the measurements at the scan body level and the landmarks chosen as controls at the level of the neighboring natural teeth. When we compared the first implant plane with the control plane, we did not identify a statistically significant correlation (*p* = 0.181), and this remained true for the second (*p* = 0.758) and third implant planes (*p* = 0.127).

We then compared the average values for all three implant planes with those for the control planes and found no statistically significant correlation (*p* = 0.198).

**Figure 8:**
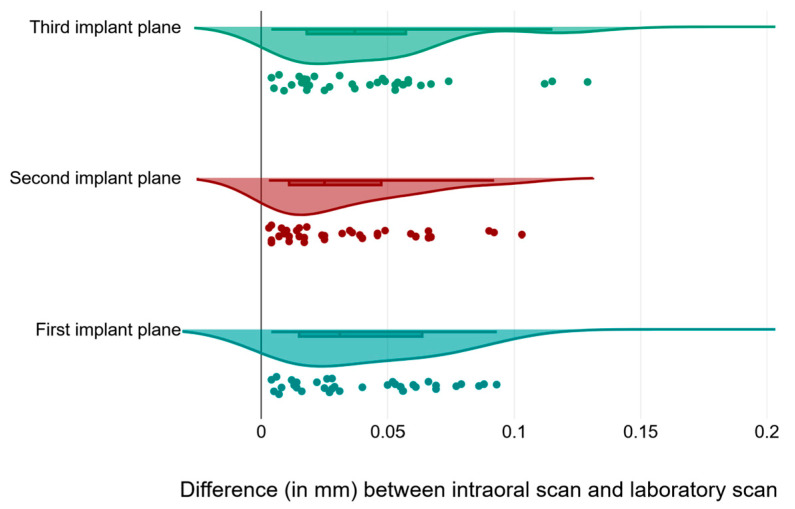
Raincloud plot visualization of measurements (expressed in mm) across different implant planes for raw data and data distribution, along with boxplots presenting the 25th and 75th percentiles along with the medians.

In order to understand the source of the difference between the measurements for the implant planes and the control planes, we plotted the values for each of the implant planes together (Figure 8) and noticed that the data had different degrees of scattering, particularly for the first and third implant planes.

**Figure 9:**
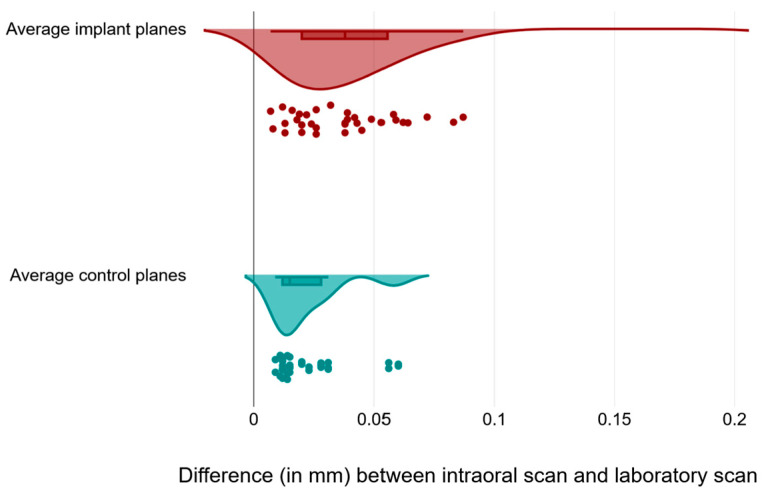
Raincloud plot visualization of measurements (expressed in mm) across implant and control planes for raw data and data distribution, along with boxplots presenting the 25th and 75th percentiles along with the medians.

We further plotted the average values for the three implant planes against those of the control plane, and we again confirmed a wider variation for the implant scans compared to the control scans (Figure 9). 

**Figure 10:**
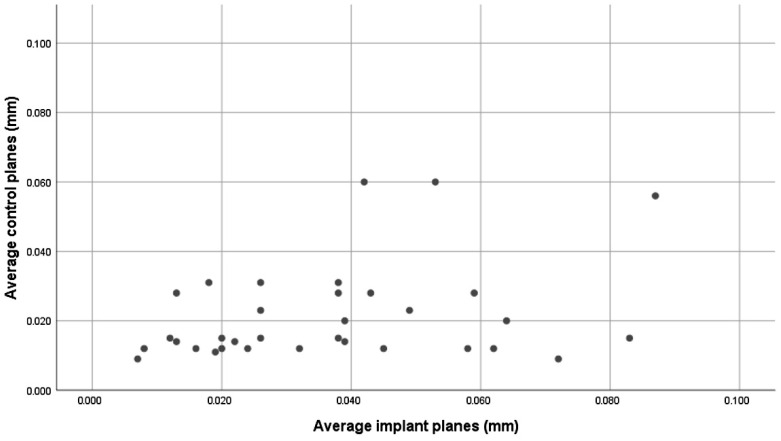
Scattering of the control measurement values (vertical axis) and implant plane values (horizontal axis).

Figure 10 shows that there was a much higher scattering of the values of the implant plane measurements compared to the controls for the neighboring natural teeth.

We calculated the difference (in mm) between the averaged implant planes and the control plane and found that it ranged from −0.018 mm to +0.267 mm, with a median of −0.011 and an IQR of (−0.001–0.031).

**Figure 11:**
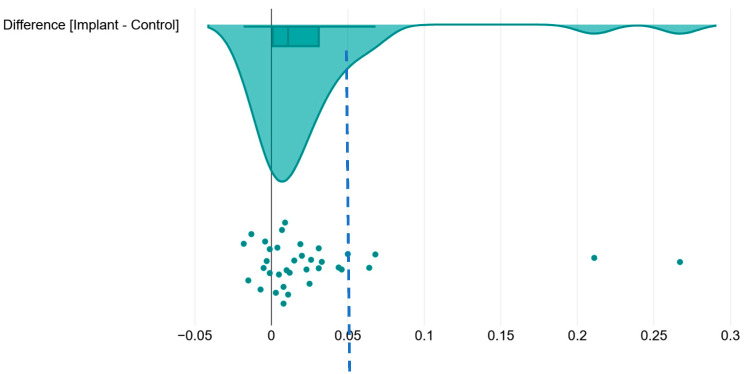
Raincloud plot visualization of the difference between the average implant level measurement and control measurement (expressed in mm) for raw data and data distribution, along with boxplots presenting the 25th and 75th percentiles along with the medians. The dashed blue line represents a theoretical clinically acceptable margin of error of 50 microns (0.05 mm).

If we consider a theoretical margin of error of 50 microns (0.05 mm) to be clinically acceptable, we notice that 4 (12.5%) out of 32 values (0.0640 mm, 0.0680 mm, 0.2110 mm and 0.2670 mm) fall outside this acceptable range (Figure 11).

## 4. Discussion

Digital dentistry has revolutionized the way dental professionals provide patient care, from diagnosis to treatment planning to new treatment approaches. In other words, the use of digital technologies in dentistry has improved accuracy and precision, as well as patient experience and communication, thus providing better treatment outcomes, enabling dental professionals to diagnose and treat patients more accurately and efficiently.

The introduction of computer-aided design/computer-aided manufacturing (CAD/CAM) technologies made it possible to manufacture implant-supported restorations through a digital workflow. The topic of the present study is not new, and there are several other studies covering this research topic. However, with intraoral scanners becoming more present in our daily clinical work, more scientific, evidence-based studies regarding their accuracy are needed. 

Making a precise implant master cast necessitates an accurate dental impression, considered until recently to be one of the most essential and time-consuming procedures in the dental office [14]. Several studies have shown that digital impressions enable reduction in working times, being time-efficient [7,14,15,16,17]. Sebastian B.M. Patzelt et al. reported that computer-aided impression making (CAIM) was substantially faster for all tested scenarios, providing a more time-efficient workflow. The results showed statistically significant differences between the durations for making digital impressions (5, 6 and 20 min) and conventional impressions (from 18 to 30 min) in three selected scenarios [14].

A meta-analysis study by de Oliveira, N.R.C. et al. showed better clinical efficiency considering impression time and time efficiency. Ten studies were included, clinical trials assessing conventional versus digital workflows for single-implant crowns being the inclusion criteria. The results showed a statistically significant reduction in time when using intraoral scanners, ranging between 6 and 20 min, compared to conventional impressions, which ranged between 11 and 28 min [18]. 

The main feature that an intraoral scanner should have is accuracy, defined as transferring the implant position correctly. An intraoral scanner should also have high trueness, i.e., it should be able to detect any detail of the clinical situation and transfer it into a digital 3D model with as high a degree of similarity to the reality as possible. Precision must also be closely considered, being defined as the ability to obtain the same value on multiple measurements [19,20,21,22].

Imburgia et al. compared the ability of four different intraoral scanners to record high-quality impressions in patients with dental implants. They analyzed two different clinical situations: a partially edentulous model with three implants and a fully edentulous model with six implants. The two models were scanned with a reference scanner (ScanRider^®^) and with four intraoral scanners (CS3600^®^, Trios3^®^, Omnicam^®^ and TrueDefinition^®^), with five scans taken for each model. The authors found significant differences in trueness among the intraoral scanners. The trueness and precision were higher in the partially edentulous clinical situation than in the fully edentulous model—a result with important clinical implications [23].

Andriessen F.S. et al. tested the accuracy of an intraoral scanner in twenty-five edentulous mandible cases. Each patient had a complete mandibular overdenture retained by two implants. The researchers performed two scans for each case: one of the scan bodies tightened on the implants intraorally and performed with the iTero intraoral scanner; the other of the implant analogs of the master casts and performed with an extraoral laboratory scanner (a Lava Scan ST scanner). The results showed that the errors regarding distance and angulation were too large to allow the manufacture of well-fitting frameworks on implants in edentulous mandibles.

Some studies have also reported contradictory results. Regarding the accuracy of digital full-arch implant impressions, in vitro studies by Pesce P. et al., Amin S. et al. and Menini M. et al. have shown better results than the previous study [24,25,26]. Amin S. et al. concluded that full-arch digital implant impressions using two scanners (True Definition and Omnicam) were significantly more accurate than conventional impressions obtained with the splinted open-tray technique [24]. 

Papaspyridakos P. et al. also evaluated the differences between the digitized conventional model and intraoral scans of thirty-six edentulous jaws and reported a 88 ± 24 μm cumulative 3D deviation between the digital casts from intraoral scans and digitized stone casts generated from conventional implant impressions [27].

In partially edentulous patients, intraoral digital implant impressions using scanning abutments offer more accuracy than the conventional open-tray/closed-tray impression technique [28].

In a study published by Nedelcu et al., the precision and fidelity of intraoral scanners were tested. The data from this study suggested precautions in the utilization of intraoral scanners by dental professionals and recommended the usage for partial edentations. Over time, the precision and fidelity of scanners have improved, allowing them to be used in complex oral rehabilitations. The results of Nedelcu’s et al. study match the results of our study [29].

Thus, many factors may have an effect on the accuracy of digital impressions, including the scan abutment design and the material type. The majority of scanning abutments commercially available are made of titanium or polyetheretherketone (PEEK), with heights between 3 and 17 mm, and can be easily adapted for specific intraoral situations [30].

Many commercial brands have developed scan bodies with different designs, geometries and surface treatments. Motel C. et al. concluded that even scan body type and scan strategies influence the quality of digital impressions of implants [31]. An experimental study by Fluegge T. et al. found that the precision of extraoral scanning of scan bodies depends on the scan body surface; usually, smooth and opaque surfaces are easier to capture than translucent or shiny ones [32]. 

Considering the data available in the literature, we can affirm that our results are partially in accordance with those of other studies, but there are also some major differences. Our study evaluated the differences between two different scanning methods of a physical model with implant analogs using an intraoral scanner (Trios 3, 3Shape, Copenhagen, Denmark) and the gold standard, a laboratory scanner (Medit Corp., Seoul, Republic of Korea). 

The study hypothesis was that there would be no statistically significant differences between intraoral scanning and classic transfer impressions taken with vinyl polysiloxane materials. The impression technique used to obtain the physical models was the same for all three cases—open-tray impression, with transfer abutments splinted with self-curing acrylic resin (Pattern Resin, GC, Tokyo, Japan) and silicon impression materials (Honigum Pro, DMG, Hamburg, Germany). All models were cast in a dental laboratory, and original implant analogs from the implant manufacturer (MegaGen, Daegu, Republic of Korea) were embedded in the cast. 

Each model was scanned with the intraoral scanner five times in order to check the consistency of the results. The laboratory scan was performed only once, using the resulting STL file as a reference scan. The superimposition of the intraoral and lab scans was performed by a trained dental technician. In order to eliminate from the statistical analysis any errors in the superimposition of the two scans, we defined two control planes—two sections located anterior and posterior to the dental implants. We thus considered that if there were differences between the positions of the scan bodies in the intraoral scan and the lab scan and the same differences were to be found in the control sections, there would be a problem of superimposition.

Our hypothesis was that the intraoral scan and the laboratory scan should be very similar, since we used the same models and the same transfer abutments. But if we look at Figure 9 and Figure 10, we can see that the results were extremely varied. 

We compared the measurements in the averaged control planes with each of the three planes at the level of each scan body and with an average of the implant measurements. These planes were described for the present study in order to cover all possible mismatches between the intraoral scan and the laboratory scan. 

The statistical analysis shows that, for all comparisons, there was no correlation between the control measurements and the implant measurements. This can be best observed in a direct comparison of the distribution of the measured values (Figure 9). We can observe from Table 1 that the distribution of the control measurement values is relatively grouped, with a median of 0.015 mm, and an interquartile range between 0.012 and 0.028, while the distribution of the study measurements was much wider. 

These differences can also be observed directly, without a statistical analysis, if we look at the data in Figure 10, which are very heterogenous. To look at an example, for one of the implants, the measured difference between two scans corresponding to plane 1: in scan 1, it was 0.78 mm (which is a very high value), while in the second scan it was 0.069 mm—almost 10 times lower. In this situation, it is very possible that this was an error of scan body positioning—either it was not screwed down completely or it was positioned differently. 

When we specifically looked at the differences between the implant measurements and the control measurements, we found that most values were within a clinically acceptable theoretical margin of error of 0.05 mm. However, in four instances (representing 12.5% of the measurements), the values fell out of this acceptable range, two (6.25%) of them being relatively close to 0.05 (0.0640 mm and 0.0680 mm, respectively), while two (6.25%) other values (0.2110 mm and 0.2670 mm) represented extreme outliers.

Other differences between intraoral scans can be caused by the scanning protocol used. Each intraoral scanning device producer makes certain recommendations regarding the scanning protocol for the dental arch in order to avoid errors when the software performs the stitching of the successive images generated by the scanner. The 3Shape company, for example, recommends first scanning the occlusal surface of all teeth for the maxillary arch, then moving to the buccal region and scanning all buccal surfaces before moving to the palatal region and scanning all palatal surfaces, followed by the palate. This way, the occlusal surface acts as a landmark for stitching the images from the buccal and palatal surfaces. If the scanning protocol is performed differently, it is possible that errors in the spatial arrangement of the dental arch will be generated. 

Even if some differences between the two scanning methods may seem large, they must be interpreted in the context of the prosthetic work that will be seated on those implants. If a single-unit crown procedure is going to be performed, these differences most likely will not have a high impact in a real clinical situation. In practice, small differences might be noticed between the computer design of the crown and the actual manufactured crown, such as too tight proximal contacts or incorrect occlusal points.

But if a splinted multiple-unit restoration is planned to be performed, the results of our study suggest caution is warranted. If the prosthetic bridge is screw-retained on the implants, the tolerance for error is close to 0. Any differences between the real position of the implants and the position of the digital analogs generated in the 3D modelling software used may determine a lack of passive fit of the prosthetic framework, with tensions transmitted to the implants. These tensions may determine the fracture of the frame (especially zirconia frames), fracture of the fixation screws, or even the fracture of the implant, in extreme cases. Also, any residual tension may determine cervical bone loss around the implants. 

By observing the results of the present study, we can also offer solutions to these potential problems in order to benefit from the multiple advantages of a digital workflow. The first solution would be to choose cemented retained restorations on custom abutments. The abutments are individually screwed on the corresponding implants, and any potential differences can be compensated by the cementing material, which also acts as a passivation for the bridge. 

If the prosthetic bridge must be screw-retained, a solution could be to cement the titanium bases intraorally instead of cementing them on a printed model with digital analogs. In order to be able to do this, the dental technician must generate a slightly larger cement space inside the crown so that there is no friction between the inside of the crown and the titanium base, and the titanium base can be cemented inside the crown intraorally. This way, the entire framework is passivated, and after the setting of the luting material it can be unscrewed, the excess cement can be removed, and it can be screwed back on the implants. This method can also be applied to the classic protocol, but it can be somewhat difficult and time consuming. However, with a digital workflow, the entire treatment time is much shorter, so one can afford to take the extra time to ensure that the delivery of the final restoration is performed in the best possible conditions. 

Our study has certain limitations. First, the scans were matched using Exocad rather than a specialized software package for STL file alignment like Geomatix. However, the fact that we defined control measurements at the level of natural teeth adjacent to the scan bodies compensated for the potential superimposition errors. Also, our results are in accordance with those of other studies, like Lee et al.’s [33] and Winkler et al.’s [34], which came to the same conclusion using slightly different methodologies: in limited restorations, intraoral scanning has sufficient accuracy, but for full-arch scans clinicians must use this method with caution. 

## 5. Conclusions

Within the limitations of the present study, it may be concluded that when it comes to single-unit implant-supported restorations, intraoral scanning may not have enough accuracy compared to the classic approach—pick-up impression with an open tray. 

However, our results show that the differences that result when scanning with an intraoral scanner may affect the quality of prosthetic work on multiple implants, especially if they are screw-retained. Nevertheless, these potential errors generated by the digital workflow can be managed by adapting the prosthetic protocol—switching to bridges cemented on custom abutments or intraoral cementation of the titanium bases for screw-retained bridges. 

In the end, even if a full digital protocol has the potential to generate some errors, knowing exactly where these errors can occur and how to fix or avoid them may provide indications for choosing this workflow, especially since it offers multiple advantages over the classic protocol both for the clinician and dental technician team and for the patient. 

## Figures and Tables

**Table 1:** Measurements (in mm) performed in the steps described in the Methods section.

	First Implant Plane	Second Implant Plane	Third Implant Plane	Averaged Implant Planes	Averaged Control Planes
Median (IQR)	0.031 (0.014–0.066)	0.025 (0.011–0.049)	0.037 (0.018–0.058)	0.038 (0.020–0.058)	0.015 (0.012–0.028)
Statistical correlation vs. control plane	r_s_(35) = 0.231, *p* = 0.181	r_s_(35) = 0.054, *p* = 0.758	r_s_(35) = 0.263, *p* = 0.127	r_s_(35) = 0.223, *p* = 0.198	N/A

Values are reported as medians (interquartile ranges) and are expressed in mm. Statistical analysis was performed using the two-tailed Spearman correlation test.

## Data Availability

All data are available upon reasonable request.
